# Variations in Swine Liver Anatomy

**DOI:** 10.1097/TP.0000000000004971

**Published:** 2024-05-24

**Authors:** Patrik Mik, Lada Eberlová, Zbyněk Tonar, Václav Liška

**Affiliations:** 1 Department of Anatomy, Faculty of Medicine in Pilsen, Charles University, Pilsen, Czech Republic.; 2 Department of Histology and Embryology, Faculty of Medicine in Pilsen, Charles University, Pilsen, Czech Republic.; 3 Department of Surgery and Biomedical Center, Faculty of Medicine in Pilsen, Charles University, Pilsen, Czech Republic.

Having read with great interest the article by Cho et al,^1^ we would like to share a few comments regarding the variability of porcine liver anatomy and the segmental nature of the liver.

**FIGURE 1.**
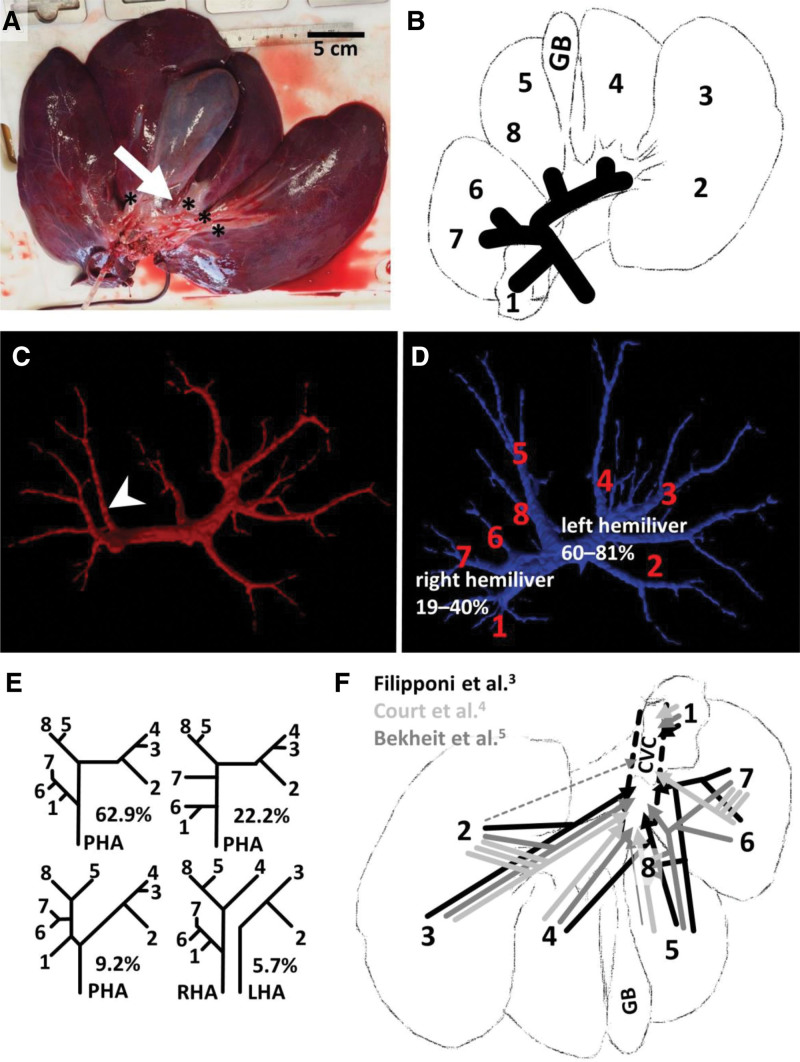
Morphological variability in porcine liver. A, In Prestice Black-Pied pigs, the quadrate lobe (white arrow) was not developed in 65% of cases.^2^ Asterisks mark PV segmental branches within a fibrous sheath. B, Depicted PV segmental branches according to Filipponi et al,^3^ the first branch of the PV goes to segment 1, the second branch is a common trunk for segments 6 and 7. The PV then curves medially and usually gives either 1 common branch or 2 separate branches for segments 5 and 8, before entering the liver parenchyma. After a short course of approximately 2.5 cm, it bifurcates into a branch for segment 2 and a common trunk for segments 3 and 4. C, Trifurcation of the PV was observed in 1 case (out of 6 studied) in our experiment.^2^ This third branch (arrowhead) supplied right medial lobe. D, The most common pattern of PV branching observed in our experiment^2^ with numbered liver segments. In the strict sense, the porcine liver can be divided in the right and left hemilivers, where the right division constitutes 19%–40% of the liver volume, and the left division constitutes 60%–81% of liver volume. Segmentation was done with the use of our freely available segmentation software LISA (Liver Surgery Analyser, available at: https://github.com/mjirik/lisa). E, Branching pattern of the PHA in domestic pig according to Filipponi et al^3^ with respective prevalence. F, Variability in the venous drainage of porcine liver into the CVC. Each depicted study is represented in corresponding color. Dashed line represents observed variability in the cited study; thin line represents smaller contribution to the drainage. Three or 4 hepatic veins have been reported. Segments 2 and 4 can drain directly to the CVC; segments 4, 5, and 8 can drain into a common trunk that forms the middle hepatic vein; alternatively, only the drainage for segments 5 and 8 can form the middle hepatic vein, whereas the left hepatic vein is formed by the drainage of segments 2, 3, and 4. A, Published with consent of Petra Kochová. C–F, Modified from Mik.^2^ CVC, caudal vena cava; GB, gall bladder; LHA, left hepatic artery; LISA, Liver Surgery Analyser (available at https://github.com/mjirik/lisa); PHA, proper hepatic artery; PV, portal vein; RHA, right hepatic artery.

Based on our research, surprisingly large interspecies variability has to be expected at the gross anatomy level of porcine liver. In domestic pigs, 3–6 anatomic liver lobes have been reported. Furthermore, in our experiments on Prestice Black-Pied pigs, the quadrate lobe was developed in 35% of cases^2^ (Figure 1A).

On the largest set of pig livers to date, Filipponi et al^3^ described different branching pattern of portal vein (PV) (Figure 1B). Furthermore, we observed PV bifurcation into the left and right branch just before entering the liver parenchyma in most cases (N = 5).^2^ However, in 1 case, we observed a PV trifurcation—the caudal branch supplied the right medial lobe (Figure 1C). In total, in our Prestice Black-Pied pigs, the right branch of the PV supplied 19%–40% of the liver, whereas the left branch 60%–81% of the liver (Figure 1D). Therefore, the volume ratio of the hemilivers was reversed when compared with humans. Concerning the branching variability of the proper hepatic artery (PHA), the branching scheme proposed by Cho et al^1^ has not yet been published. The most common branching pattern of the porcine PHA (63%) is shown in the Figure 1E. Liver drainage is usually assumed to be mediated by 3 hepatic veins with separate drainage of the caudate lobe/process; however, a separate drainage of segment 2 directly into the caudal vena cava has also been reported. Court et al^4^ reported 4 hepatic veins in agreement with Cho et al.^1^ Furthermore, we summarize the published variability in the branching pattern of the segmental veins in the Figure 1F.^3,4,5^

Although the Brisbane terminology has been adopted worldwide, we have 3 concerns regarding the definition of the liver segment. First, the borders of the segments are considered planar, whereas they are in fact undulated and form interdigitations. Second, the correlation of branching liver vascular beds is assumed. However, it has been demonstrated that individual liver vascular beds do not copy each other in the pattern of branching. Third, the definition of liver segment is circular, for the liver segment is usually considered as a part of the liver that is supplied/drained by its segmental vessel. On the other hand, the definition of the liver segment by the order of the generation of vessel branches is not satisfactory too; the liver has been reported to have 24 second-order PV branches; therefore, the grouping of these branches is arbitrary.

The importance of pigs as a source of organs for the training of surgical techniques and xenotransplantation is indisputable, as is the research on the morphology of porcine liver vessels. In our commentary, we highlighted the already proven, significant inter-individual and breed-specific variability. Therefore, we strongly recommend computed tomography imaging before any pig liver resection.
